# Coronary artery calcium testing in low-intermediate risk symptomatic patients with suspected coronary artery disease: An effective gatekeeper to further testing?

**DOI:** 10.1371/journal.pone.0240539

**Published:** 2020-10-13

**Authors:** Tahir Mahmood, Michael D. Shapiro

**Affiliations:** 1 Center for Preventive Cardiology, Knight Cardiovascular Institute, Oregon Health & Science University, Portland, OR, United States of America; 2 Center for Prevention of Cardiovascular Disease, Section on Cardiovascular Medicine, Wake Forest University School of Medicine, Winston-Salem, NC, United States of America; University of Perugia, ITALY

## Abstract

Computed tomography for quantification of coronary artery calcium (CAC) is a simple non-invasive tool to assess atherosclerotic plaque burden. CAC is highly correlated with coronary atherosclerosis and is a robust predictor of cardiovascular outcomes. Recently, the 2018 ACC/AHA Cholesterol Guidelines endorsed the use of CAC scores in asymptomatic, intermediate risk individuals where the decision to initiate stain therapy is uncertain. However, whether quantification of CAC may play a role in the assessment of symptomatic individuals remains a matter of debate. In this review, we examine the evidence for the use of CAC in low-intermediate risk patients with chest pain. This appraisal places a particular focus on the growing body of literature supporting the negative predictive value of a CAC score of zero to rule out significant coronary artery disease in those without high-risk features. We also evaluate current guidelines, limitations, and future research directions for CAC scoring in this important subgroup of patients.

## Introduction

**Fig 1:**
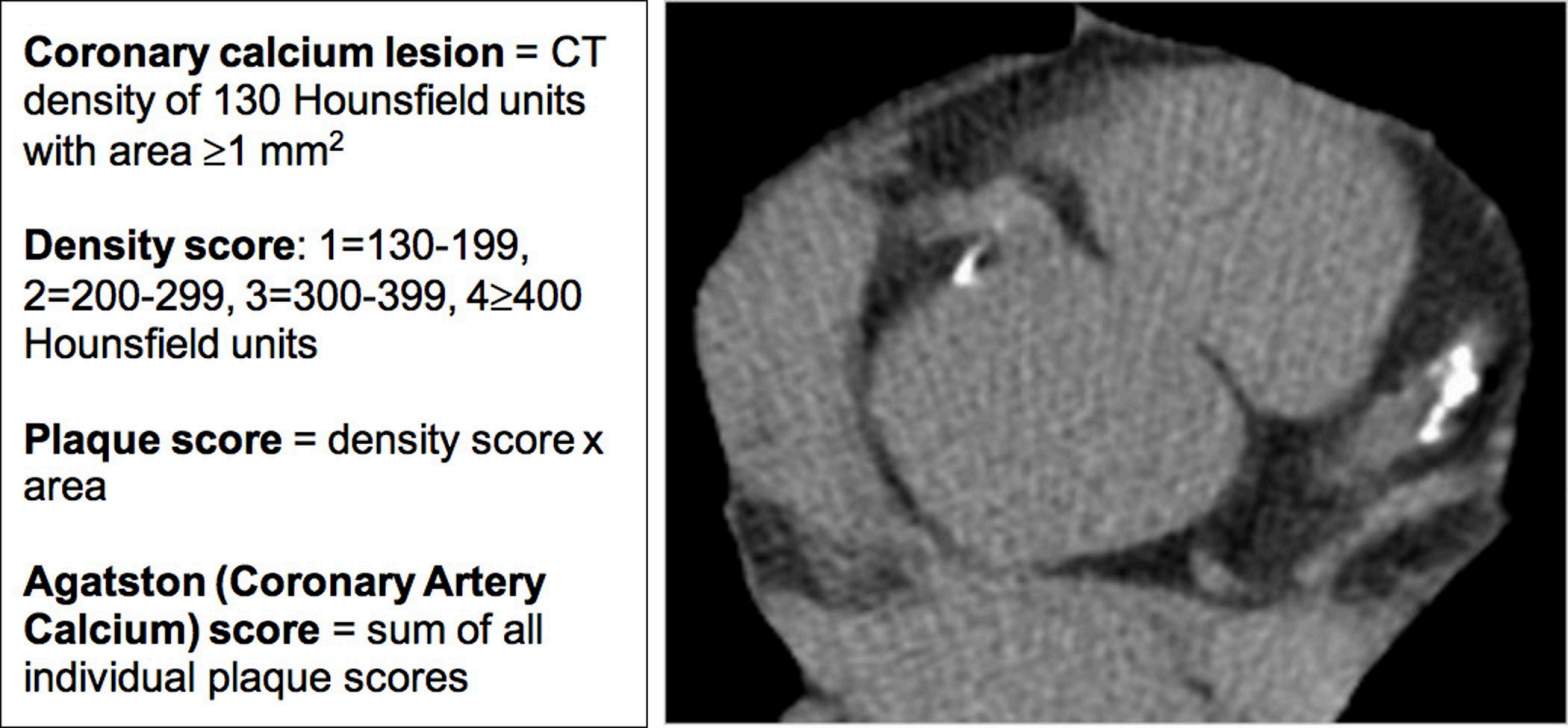
CAC scoring methodology and example image. CAC scoring methodology is depicted on the left. The Agatston (CAC) score is what is used in clinical practice. On the right is an example of a single slice (image) from a CAC scan that demonstrates calcification in the left anterior descending and right coronary arteries. CAC, coronary artery calcium; CT, computed tomography.

Coronary artery calcium (CAC) scoring has emerged as an important tool to refine atherosclerotic cardiovascular disease (ASCVD) risk. Vascular calcification was originally thought to be an unmodifiable process of aging. More recently, studies have demonstrated that development of calcific atherosclerosis is a slow but active process involving the complex interplay between apolipoproteins, oxidized lipids, inflammation, and osteogenic factors [1]. Vascular calcification also represents an opportunity to use imaging for detection of subclinical atherosclerosis. CAC scoring is a non-invasive imaging method developed three decades ago by Agatston *et al*. [2] to quantify calcified plaque burden in the epicardial coronary arteries. Initially developed using electron-beam computed tomography (CT), current practice employs modern multidetector CT [3]. Nonetheless, Dr. Agatston’s original scoring system based simply on calcium area and density remains the gold standard for CAC quantification (Fig 1) and the basis for standardized scoring categories [4, 5] as well as percentiles distributed by age, gender, and ethnicity [6]. Detection and quantification of CAC is important for both diagnosis and prognosis of coronary artery disease (CAD) and holds the promise to ultimately improve outcomes in cardiovascular disease, which remains the leading cause of death worldwide [7].

CAC scoring has been extensively studied in asymptomatic individuals for primary prevention of ASCVD. In patients free of clinical ASCVD, not only is CAC scoring highly specific for coronary atherosclerosis but it is also a strong predictor of cardiovascular events [1, 4]. However, given recent changes in quantitative risk assessment and thresholds for initiation of statin therapy for primary prevention, CAC quantification has become not only useful in detecting clinically significant disease but also discovering its absence. Large studies in asymptomatic individuals demonstrate that a CAC score of zero is associated with a reassuringly low 10-year cardiovascular event rate of ~1% [8, 9]. This phenomenon, coined ‘power of zero’ [10], has since been endorsed in the most recent 2018 ACC/AHA Cholesterol Guidelines [11]. Specifically, in asymptomatic patients at intermediate risk for fatal and non-fatal myocardial infarction (MI) and stroke (10-year ASCVD risk score of 7.5–20%) as quantified by the Pooled Cohort Equations [12], a clinician should consider CAC measurement if the decision to start a statin is uncertain. A CAC score of 0 functionally “de-risks” an individual to a lower risk category and often facilitates the safe delay or deferral of statin therapy.

In this review, we explore the potential role and evidence for CAC scanning in low-intermediate risk patients with chest pain. In addition to ‘de-risking’ asymptomatic individuals, a CAC score of zero has been studied as a means to exclude significant CAD in low-intermediate risk symptomatic patients. In this context, CAC scanning may serve as a gatekeeper to additional testing, with the absence of CAC eliminating the need for further cardiovascular imaging [13]. The use of CAC testing in low-intermediate risk patients for evaluation of chest pain should be cost-effective but it is not yet guideline recommended [14–17]. Accordingly, the purpose of this review is to discuss the growing evidence regarding the potential role for CAC testing in select patients who require an evaluation to rule out significant CAD.

### Imaging to exclude CAD in low-intermediate risk symptomatic patients

Choosing an imaging modality for a patient with chest pain is dependent on the pre-test probability for CAD. Chest pain with high pre-test probability for CAD warrants invasive coronary angiography for diagnosis and potential intervention [14, 16]. This review focuses on the low-intermediate risk patient population. Low-intermediate risk symptomatic patients include those without known ASCVD who exhibit chest pain that is atypical for angina. In the setting of acute chest pain, low-intermediate risk individuals have relatively new onset symptoms with a non-ischemic electrocardiogram (ECG) and a first troponin that is normal. Such patients may benefit from a highly sensitive, non-invasive (e.g., gatekeeper) test to potentially obviate the need for more advanced diagnostics, which have their own associated risks and costs.

Stress testing, in its myriad forms, has long been the initial non-invasive diagnostic test to evaluate suspected CAD in symptomatic individuals. However, recent trends demonstrate that the overwhelming majority of stress myocardial perfusions scans (MPS) are normal. Rozanski *et al*. [18] demonstrated in a large population that the rate of abnormal and ischemic stress MPS in 2009 was 8.7% and 5%, respectively. Consequently, anatomical imaging has garnered interest as a more cost-effective initial test in low-intermediate risk patients with suspected CAD [13, 19]. In this setting, coronary computed tomography angiography (CCTA) was compared to stress testing in the randomized trial PROMISE (Prospective Multicenter Imaging Study for Evaluation of Chest Pain) [20]. No significant differences in clinical outcomes were observed between those who underwent anatomic versus functional testing after a median follow up of 25 months. Furthermore, the incidence of positive stress tests was quite low (11.7%) in this symptomatic population with a mean 53% pretest probability for obstructive CAD. The SCOT-HEART (Scottish COmputed Tomography of the HEART) trial randomized patients with stable chest pain to standard care plus CCTA or standard care alone [21]. The primary endpoint of death from coronary heart disease or nonfatal MI was ultimately found to be significantly decreased in the CCTA group after 5 year follow up (2.3% versus 3.9%), likely related to more accurate CAD diagnosis leading to more appropriate use of preventive therapies [22]. Indeed, current guidelines support the use of CCTA in appropriate lower risk symptomatic patients [14–17]. Evidence also suggests that CCTA may significantly lower costs in the emergency department (ED) [23].

### Intrinsic qualities of CAC scan as gatekeeper test

While CAC testing and CCTA are both CT based anatomic imaging techniques, they have important differences (Table 1). CAC scanning is less technical than CCTA, providing it with a potential advantage over CCTA as a gatekeeper test [13]. Beyond its relatively straightforward methodology (Fig 1), CAC scoring has many intrinsic test qualities that embody a gatekeeper. It was, after all, initially intended as a screening test in primary prevention populations, making its possible transition to a sensitive diagnostic test rather natural. CAC testing is widely available and does not require the use of iodinated contrast agents. Nuclear studies are associated with a significantly higher radiation dose (5–41 mSv) compared to both CAC and CCTA [24]. The images for CAC scanning are rapidly obtained (<10 second breath hold with <15-minute room time) and results can be interpreted quickly in order to inform further diagnostic decisions. Lastly, CAC scanning is the most economically feasible of the diagnostic cardiac imaging modalities, typically costing less than $100. With time and budget constraints as well as contraindications inherent to clinical testing, CAC scoring possesses several advantages that may allow for the responsible stewardship of medical resources in lower risk patients with chest pain.

**Table 1:** Important contrasts between CAC and CCTA.

	CAC	CCTA
**Intravenous contrast**	No	Yes
**Use of heart rate reducing medication**	No	Yes
**Post-processing period**	Faster than CCTA	Slower than CAC
**Radiation (representative effective dose value)** [24]	1 mSv	3 mSv

CAC, coronary artery calcium; CCTA, coronary computed tomography angiography; mSv, milliSievert.

### Current CAC guidelines

Clinical practice guidelines that evaluate the utility of CAC scoring in symptomatic patients are detailed in Table 2. Guidelines have yet to endorse the use of CAC scanning for symptomatic patients, including those at lower risk for CAD. As mentioned previously, the 2018 AHA/ACC Cholesterol Guidelines endorse CAC testing in asymptomatic, intermediate risk individuals when the decision to initiate statin therapy is uncertain [11]. There remains no guideline recommendation to use CAC assessment in patients with established ASCVD.

**Table 2:** Clinical practice guidelines that evaluate the utility of CAC scoring in symptomatic patients.

Guideline	Recommendation
2013 ACCF multimodality appropriate use criteria for the detection and risk assessment of stable ischemic heart disease [14]	Rarely appropriate: Calcium scoring in symptomatic patients with low, intermediate, or high pre-test probability for coronary artery disease
2012 ACCF/AHA guideline for the diagnosis and management of patients with stable ischemic heart disease [15]	Class IIb: For patients with a low to intermediate pretest probability of obstructive ischemic heart disease, non-contrast cardiac CT to determine the CAC score may be considered
2015 ACR/ACC appropriate utilization of cardiovascular imaging in emergency department patients with chest pain [16]	Coronary calcium scoring was not considered by the rating panel because there are few data on coronary calcium scoring using multidetector CT hardware in patients who present to the emergency department in whom acute coronary syndrome is the leading differential diagnosis
2013 ESC guidelines on the management of stable coronary artery disease [17]	Class III: Coronary calcium detection by CT is not recommended to identify individuals with coronary artery stenosis

ACC, American College of Cardiology; ACCF, American College of Cardiology Foundation; ACR, American College of Radiology; AHA, American Heart Association; CAC, coronary artery calcium; CT, computed tomography; ESC, European Society of Cardiology.

### The interplay between CAC testing and stress myocardial perfusion imaging

Anatomical testing (such as CAC) and stress testing assess fundamentally different features of CAD. While CAC testing quantifies the amount of calcific atherosclerosis in the coronary arteries, stress testing evaluates the presence and extent of demand ischemia, at-risk myocardium, and infarction. There has been interest in integrating anatomical and physiologic testing to obtain the most complete assessment of the coronary arteries. In this regard, combining CAC with stress MPS has demonstrated significant promise in improving prognostic and diagnostic value [25, 26]. This review focuses on quantification of CAC as the first line test (prior to or instead of stress testing) for evaluation of individuals with low-intermediate risk chest pain. Thus, one must understand the ability of CAC to predict ischemia and cardiac events compared to stress MPS.

Large studies, including two meta-analyses, comparing a CAC score of zero and stress MPS in symptomatic patients are presented in Table 3 [8, 27–31]. These studies demonstrate two seemingly disparate, but overall, reassuring findings. First, a CAC score of zero does not appear to completely rule out significant myocardial ischemia. While some studies demonstrate rates of ischemia close to 1% associated with a CAC of 0, other studies exhibit rates of ischemia as high as ~6–7%. Second, despite these rates of ischemia, incident hard cardiovascular events in patients without CAC were very low (Table 3). Of particular interest are the analyses that compared outcomes between abnormal and normal stress MPS in patients without demonstrable CAC. Paradoxically, both studies demonstrated trends of increased cardiovascular events in patients with normal stress MPS compared to those that demonstrated ischemia [27, 31]. In patients with CAC = 0, Engbers *et al*. [31] found that 12% of such individuals exhibited abnormal stress MPS with 67% demonstrating small perfusion defects (defined as <10% of the left ventricular myocardium) and 4% demonstrating large defects (≥20% of left ventricular myocardium). Low annualized event rates were found in patients with CAC = 0, irrespective of whether the MPS was normal or abnormal (0.61% vs 0.41%, respectively). Mouden *et al*. [29] also found that 12% of patients without demonstrable CAC had abnormal stress MPS. All 102 of these patients were ultimately ruled out for obstructive disease via CCTA or invasive angiography and there were no coronary events in all 868 patients without CAC after 1.5 years. In a study exclusively performed in the ED [28], CAC = 0 was found in 61% of patients presenting with stable chest pain without history of CAD as well as a normal initial troponin and non-ischemic ECG. Only 2 out of 625 of these patients had a cardiac event at 7-month follow-up. Moreover, both of these patients were considered to have an event due to abnormal subsequent troponin levels during their index hospitalization, though their stress MPS were normal. A more recent meta-analysis of acute chest pain in the ED including 3,556 patients from 8 studies confirms these findings [32]. Specifically, the pooled prevalence of CAC = 0 was 60%. With a median follow-up of 10.5 months, the pooled annualized rate of major adverse cardiovascular events was 0.8% in patients without demonstrable CAC compared to 14.6% in patients with CAC present. Overall, it is reassuring that event rates in those with CAC = 0 remain quite low despite results suggestive of ischemia on stress MPS [13].

**Table 3:** Studies evaluating outcomes of symptomatic patients who underwent stress myocardial perfusion imaging and had a coronary artery calcium of zero.

Study	Population	Outcomes	Percentage of participants with CAC of zero	Outcomes in participants with CAC of zero
Rozanski *et al*., 2007 [27]	1,153 patients referred for CAC scan and stress MPS within 6 months of each other (49% symptomatic)	Cardiac death and MI after mean follow-up of 32 months	22%	1.2% with evidence of ischemia with 0 cardiac death/MI events.
				Nonischemic patients with 0.2%/year annualized cardiac death/MI rate.
Sarwar *et al*., 2009 [8]	Meta-analysis: 7 studies including 3,924 symptomatic patients	Cardiac events (with all studies including cardiac death and MI) over mean follow-up of 42 months	23%	1.8% had event.
	8 studies including 3,717 patients who underwent CAC with stress MPS	Evidence of ischemia on stress MPS	26%	7% with evidence of ischemia.
Nabi *et al*., 2010 [28]	1,031 prospectively enrolled stable patients presenting to the ED with chest pain of uncertain cardiac cause underwent both CAC and stress MPS within 24 hours of ED admission	Cardiac events defined as cardiac death and ACS (MI or unstable angina pectoris) during index hospitalization or mean follow-up of 7 months	61%	0.8% with abnormal stress MPS. 0.3% had event.
Mouden *et al*., 2013 [29]	3,501 symptomatic stable patients without known CAD underwent prospective simultaneous stress MPS and CAC	Events defined as coronary revascularization, nonfatal MI, and death with median follow-up of 17 months	25%	12% with abnormal stress MPS.
				No coronary events.
Bavishi *et al*., 2016 [30]	Meta-analysis: 6 studies including 2,123 patients (mixture of asymptomatic and symptomatic) who underwent CAC and stress MPS	Frequency of inducible ischemia on stress MPS	23%	6.6% prevalence of ischemia.
Engbers *et al*., 2016 [31]	4,897 symptomatic stable patients without known CAD underwent prospective stress MPS combined with CAC	MACE defined as late revascularization, nonfatal MI, and all-cause mortality with median follow-up 940 days	27%	12% with abnormal stress MPS had 0.41% annual event rate.
				0.61% annual event rate in patients with normal stress MPS.

CAC, coronary artery calcium; CAD, coronary artery disease; ED, emergency department; MACE, major adverse cardiovascular event; MI, myocardial infarction; MPS, myocardial perfusion scan.

### The interplay between CAC testing and coronary computed tomography angiography

CCTA is the initial test of choice in select patients with chest pain and suspected CAD [14–17]. While both CAC and CCTA provide information on overall plaque burden, CCTA can provide more precise information on plaque composition/morphology and evaluation of luminal narrowing. CCTA can thus evaluate the presence of non-calcified atherosclerotic plaque and whether a CAC score of zero reliably excludes obstructive CAD (≥50% stenosis).

CCTA has been extensively studied in symptomatic individuals. Fortunately, multiple large studies of CCTA, including several randomized controlled trials (Table 4) [33–37], also included analyses of CAC. The CONFIRM (Coronary CT Angiography Evaluation for Clinical Outcomes: An International Multicenter) registry enrolled over 10,000 symptomatic patients without known CAD at the time of CCTA [33]. These patients underwent concurrent CAC testing and cardiovascular events were followed for over 2 years. The absence of CAC effectively ruled out obstructive CAD (NPV 96.5% for ≥50% stenosis; 98.6% for ≥70% stenosis) and major adverse cardiovascular events occurred in <1%. Of note, participants with a CAC of zero but the presence of obstructive CAD exhibited a significantly higher major adverse cardiovascular event rate (all-cause mortality, nonfatal MI, late coronary revascularization) of 3.9%, though this was predominantly driven by late coronary revascularizations (5 of the 7 patients with events). Of note, there were no deaths observed in this subgroup.

**Table 4:** Studies evaluating outcomes of symptomatic patients who underwent coronary computed tomography angiography and had a coronary artery calcium of zero.

Study	Population	Outcomes	Percentage of participants with CAC of zero	Outcomes in participants with CAC of zero
Villines *et al*., 2011, CONFIRM [33]	10,037 symptomatic patients without known CAD who underwent CCTA with CAC	All-cause mortality and the composite endpoint of mortality, MI, or late coronary revascularization after median follow-up of 2.1 years	51%	0.4% all-cause mortality. 0.9% for the composite endpoint.
Lubbers *et al*., 2016, CRESCENT [34]	242 patients with stable angina were prospectively randomized to tiered cardiac CT protocol with CAC and subsequent CCTA if CAC present or >70% pre-test probability	Composite endpoint of all-cause mortality, non-fatal MI, major stroke, unstable angina pectoris with objective ischemia and/or requiring revascularization after mean follow-up of 1.2 years	41%	No events in all 98 patients ruled out for CAD based on zero CAC.
Budoff *et al*., 2017, PROMISE [35]	4,209 patients with stable chest pain or dyspnea and intermediate pre-test probability for obstructive CAD randomized to CCTA with CAC	Primary endpoint of all-cause death, MI, or unstable angina hospitalization after median follow-up of 26.1 months	35%	1.4% for the primary endpoint.
Lubbers *et al*., 2018, CRESCENT-II [36]	130 patients with stable angina were prospectively randomized to tiered cardiac CT protocol with CAC and subsequent CCTA if CAC present or >80% pre-test probability	Major adverse events including death, nonfatal MI, unstable angina, urgent revascularization, and stroke after mean follow-up of 8 months	39%	No events in all 45 patients ruled out for CAD based on zero CAC
Williams *et al*., 2019, SCOT-HEART [37]	1,769 patients with stable chest pain in outpatient clinic were randomized to CCTA with CAC	Primary clinical endpoint of coronary heart disease death or nonfatal MI after median follow-up of 4.7 years	39%	Primary clinical endpoint of ~1% (approximated from Fig 4)

CAC, coronary artery calcium; CCTA, coronary computed tomography angiography; CAD, coronary artery disease; CRESCENT, Computed Tomography vs. Exercise Testing in Suspected Coronary Artery Disease; CONFIRM, Coronary CT Angiography Evaluation for Clinical Outcomes: An International Multicenter Registry; MI, myocardial infarction; PROMISE, Prospective Multicenter Imaging Study for Evaluation of Chest Pain; SCOT-HEART, Scottish COmputed Tomography of the HEART Trial.

The PROMISE study was a prospective randomized controlled trial that evaluated individuals with stable chest pain or dyspnea plus an intermediate pre-test probability for obstructive CAD [20]. Quantification of CAC was performed in over 4,000 of trial participants [35]. Obstructive CAD was very uncommon in patients with zero CAC. Specifically, in patients with CAC = 0, 15/1457 patients had 50–70% stenosis on CCTA and 7/1457 patients had >70% stenosis on CCTA (NPV 99.8% for ≥50% stenosis; 99.9% for ≥70% stenosis). Over a 2-year follow-up, major adverse cardiovascular events (all-cause death, MI, unstable angina hospitalization) occurred in 1.4% of patients without CAC, which was a lower rate than those randomized to the stress-testing arm who had normal results (2.1%).

The CRESCENT (Computed Topography vs. Exercise Testing in Suspected Coronary Artery Disease) and CRESCENT-II studies were prospective, randomized controlled trials that employed a tiered testing approach [34, 36]. Specifically, quantification of CAC served as the gatekeeper to additional testing. In the CRESCENT trial [34], 350 participants with stable angina were prospectively randomized to either tiered cardiac CT or stress testing. Patients randomized to CT first underwent CAC scanning. If CAC was absent, participants did not undergo additional testing unless the pre-test probability for obstructive CAD was determined to be >70%. All patients with CAC = 1–400 underwent CCTA and stress testing or invasive angiography was performed if CAC>400. Of the 242 participants assigned to the tiered cardiac CT protocol, obstructive CAC was excluded in 98 subjects through a CAC score of zero, and none of these patients sustained cardiovascular events or required further testing after 1 year. Importantly, the tiered cardiac CT protocol appeared cost-effective, resulting in 16% less cumulative diagnostic costs and lower amounts of downstream testing (25% vs 53%) compared to the stress-testing arm. The CRESCENT-II trial yielded similar results [36]. The tiered cardiac CT protocols were similar in both trials, though one notable difference in CRESCENT-II was the requirement for a higher pre-test probability (>80%) for obstructive CAD in order to require CCTA even if there was zero CAC. The mean pre-test probability for obstructive CAD was 54% in the overall study population. In total, 130 patients were randomized to the tiered cardiac CT protocol of which 45 subjects had obstructive CAD excluded by virtue of a CAC score of 0. Again, no participants with CAC = 0 experienced major adverse cardiovascular events after a mean follow-up of 8 months. One patient presented later with acute chest pain but ultimately underwent invasive coronary angiography that excluded obstructive CAD.

A secondary analysis of the SCOT-HEART trial evaluated whether coronary artery plaque characteristics were associated with clinical outcomes in patients with stable chest pain [37]. Specifically, CCTA was performed in 1,769 patients and a post-hoc analysis investigated whether adverse plaque characteristics (positive remodeling, low attenuation, spotty calcification, napkin ring sign) predicted coronary death and nonfatal MI after a 5-year follow-up. CAC burden was also included in the analysis. Presence of adverse plaque characteristics tripled the risk (hazard ratio = 3.01) of major adverse cardiovascular events. However, advanced atherosclerotic plaque characterization did not provide incremental prognostic information as multivariable analysis revealed that the only independent risk predictor was the CAC score (hazard ratio = 1.17; p = 0.011). Furthermore, no CAC was found in 39% of the study population and these participants experienced a very low incidence of events (~1%). The investigators did find that adverse plaque characteristics on CCTA provided prognostic information in patients with a low CAC burden. In patients with CAC<100, adverse plaque significantly increased the risk of events compared to those without adverse plaque (hazard ratio = 3.38; p = 0.03). While this observation suggests that CCTA may be useful in select symptomatic patients with mild CAC (e.g., CAC score 1–100), it does not challenge the fact that very low event rates are observed in patients without CAC.

Several other studies have highlighted the value of a CAC of 0 in patients with chest pain who underwent concurrent CCTA. Bittner *et al*. [38] examined the ability of the absence of CAC to rule out acute coronary syndrome (ACS) in patients presenting to the ED with acute chest pain. A total of 826 consecutive patients were included in this observational study. Participants were without known CAD and had negative initial cardiac serum biomarkers and a non-ischemic ECG. In total, 444 subjects (54%) were found to have zero CAC and only 2 patients were diagnosed with ACS during the index hospitalization (NPV = 99.5%). Obstructive CAD was also very rare in patients without CAC (NPV 99.5% for ≥50% stenosis; 99.8% for ≥70% stenosis). Additionally, in a separate analysis from the same study, zero CAC was combined with the clinical Thrombolysis In Myocardial Infarction (TIMI) risk score. In 328 patients with zero CAC and a TIMI score of 0, obstructive disease and ACS were virtually ruled out (NPV 99.7% for ≥50% stenosis; 100% for ≥70% stenosis; 100% for ACS). Mittal *et al*. [39] evaluated the prevalence of obstructive CAD and cardiovascular outcomes in patients with stable chest pain or dyspnea with a CAC score of 0. This observational study included 3,914 patients who underwent CAC scoring; 2,730 of these patients also underwent CCTA. In the patients who underwent both CAC and CCTA, the absence of CAC was found in 52% and was associated with a very low prevalence of obstructive disease (NPV 98.3% for ≥50% stenosis; 99.5% for ≥70% stenosis). All patients with >50% stenosis underwent stress imaging or invasive angiography and flow limiting stenosis was only found in 4 of 24 patients. Moreover, all-cause mortality over a mean of 5.2 years of follow-up was low in those without CAC. Their annualized death rate was 0.3% and none of them succumbed to a coronary event. Perhaps most intriguing was the finding that the presence of non-calcified plaque did not affect survival in patients without CAC (p = 0.98), once again suggesting that a CAC of 0 likely obviates the need for additional testing. Another recent observational study also examined cardiovascular outcomes in symptomatic patients with zero CAC and non-calcified plaque [40]. A total of 1,753 patients with stable angina prospectively underwent CAC scanning; zero CAC was found in 52.2% (n = 915). Of the 751 patients with zero CAC who also underwent same day CCTA testing, non-obstructive disease (<50% stenosis) was found in 8.4% with minimal evidence of obstructive disease (NPV 98.1% for ≥50% stenosis). The incidence of MACE (cardiac death, non-fatal MI, non-elective revascularization) in patients without CAC was low despite evidence of non-calcified plaque. Over a median follow-up of 2.2 years, MACE only occurred in 5 patients (0.6%) with zero CAC, none of which were a coronary death. Of these 5 patients, 3 had normal coronary arteries on CCTA, 1 had obstructive disease on CCTA, and 1 did not undergo CCTA. Reassuringly, no MACEs occurred in zero CAC patients with non-obstructive disease on CCTA.

A recent large meta-analysis evaluated the ability of CAC scoring to predict cardiovascular events in stable, symptomatic patients [41]. This analysis included 19 observational studies and 34,041 subjects. Notably, studies involving patients in the ED setting were not included. Events were recorded over a follow-up range of 17 to 82 months. The prevalence of patients with zero CAC ranged from 11–63% between all of the studies. Cardiovascular events occurred in only 1.18% (n = 158) of participants without CAC. Annual event rates per 100 patients with zero CAC ranged from 0–3.64, with all but 3 studies demonstrating an annual event rate <1%. Not surprisingly, presence of CAC dramatically increased cardiovascular risk. The pooled random effects relative risk ratio for cardiovascular events for CAC>0 vs CAC = 0 was 5.71 (95% CI: 3.98–8.19).

### Implications of CAC = 0 in symptomatic patients

As reviewed above, the evidence suggests that the absence of CAC can serve as a gatekeeper in symptomatic patients with suspected CAD. Specifically, it appears that clinicians can safely use a non-contrast CAC scan as the initial imaging evaluation for symptomatic patients with low to intermediate pre-test probability for CAD. The absence of CAC would eliminate the need for further cardiac testing. Discovery of any CAC would necessitate additional coronary assessment. Moreover, a positive CAC score would likely provide additional prognostic information to whatever additional testing is pursued, such as stress imaging, through the identification of subclinical disease. Naturally, using CAC in this context is still grounded on clinical acumen as, for example, this would determine the difference between intermediate and high pre-test probability and thus the choice of the first diagnostic test.

Application of the ‘power of zero’ in symptomatic individuals has the potential to safely triage a substantial number of patients as well as reduce costs. The ideal gatekeeper characteristics of CAC scanning should allow for more widely available testing with less contraindications, radiation harm, and financial burden. Our review of the literature demonstrates that an estimated 22–61% (Tables 3 and 4) of lower risk patients would have a zero CAC score. Placing CAC as the initial test for evaluation of low-intermediate risk chest pain would frequently obviate the need for more expensive testing. It may also have the effect of reducing downstream testing, as already shown with CAC testing in asymptomatic individuals [42]. Accordingly, costs are likely to be reduced with this approach. A glimpse of potential cost savings can be gleaned from the United Kingdom’s experiences since the introduction of the updated 2010 National Institute for Health and Care Excellence (NICE) guideline for chest pain of recent onset. The guideline recommended CAC scoring as the first-line diagnostic investigation in symptomatic patients with low pre-test probability of CAD. In 2016, the NICE guidelines removed CAC scoring as the initial test for evaluation of recent onset chest pain and replaced it with CCTA [43]. Nonetheless, cost analyses of the previous guideline that integrated CAC testing into clinical care, including a large analysis of almost 5,000 patients, demonstrated significant reductions in downstream testing and cost for evaluation of recent onset chest pain [44]. In fact, CAC was more cost effective despite the fact that it was compared to the pre-2010 guidelines that placed more emphasis on the less costly stress ECG. One would expect similar cost-effectiveness when utilizing CAC scanning as a gatekeeper in symptomatic patients, especially given its affordability compared to other cardiac imaging modalities. The economic implications of the gatekeeper role are of paramount importance as it functions to safely and effectively provide medical resources to patients while returning the optimum value to the healthcare system. In fact, the cost benefits of CAC testing may be the most profound impact of the ‘power of zero’ in symptomatic patients.

### Limitations and considerations of CAC testing in symptomatic patients

Using zero CAC as a gatekeeper in symptomatic patients is not without potential concerns. One primary concern is the lack of a large, prospective randomized controlled trial. However, there is robust observational data as well as the smaller prospective randomized, controlled CRESCENT trials [34, 36] where CAC = 0 served as a successful gatekeeper. Clinicians may be concerned that zero CAC does not reliably exclude mild ischemia, though as previously discussed, the presence of myocardial ischemia in the absence of CAC did not hold prognostic significance. Moreover, the finding of CAC = 0 virtually rules out ACS [38]. Another commonly held concern relates to the fact that not all coronary plaques contain calcium. Moreover, calcific atherosclerosis is thought to be a late process and could represent plaque stability, which may explain why statin therapy paradoxically increases CAC [45]. The fear is that CAC scanning can potentially miss early non-calcified atherosclerosis, particularly in younger patients with a family history of premature ASCVD. This subgroup may benefit more from CCTA. Nevertheless, analysis from the SCOT-HEART trial [37] demonstrated that plaque characteristics (such as low attenuation plaque that is not visualized on a CAC scan) did not independently predict events over the CAC score. Non-calcified plaque also did not impact survival in patients without CAC in a large observational analysis [39].

Longer-term outcome data are also desired. While zero CAC appears to confer a low risk of events in the short to intermediate term, longer term outcomes are less clear, though some patients have been followed up to 13 years without coronary events [39]. Lastly, in regard to anatomical testing, there may be concerns about the limited amount of information that CAC scanning provides in comparison to CCTA. The ability of CCTA to provide detailed information on non-calcified plaque, plaque characteristics, and luminal narrowing is attractive and may impact patient and clinician behavior. While this is a common default position, robust data to substantiate this perspective is lacking and remains an active area of investigation.

### Future research directions

It is likely that more prospective outcome data from randomized controlled trials will be required in order for CAC to be accepted as a gatekeeper in symptomatic patients that require evaluation for CAD. In this regard, the ACCURATE (Assessment of Patients With suspeCted Coronary Artery Disease by Coronary calciUm fiRst strATegy vErsus Usual Care Approach (NCT03972774)) trial is currently recruiting subjects. The ACCURATE trial will enroll over 2,000 symptomatic participants with suspected CAD and will formally evaluate CAC as a gatekeeper test. Patients who have a CAC≤1 will be randomized to either cardiac positron emission tomography stress testing or medical management alone. Cardiovascular outcomes and cost-effectiveness of this strategy will be assessed over 5 years. Another ongoing trial that should be completed in the near future is the DISCHARGE (Diagnostic Imaging Strategies for Patients With Stable Chest Pain and Intermediate Risk of Coronary Artery Disease (NCT02400229)) trial. While CAC is not prospectively placed in a gatekeeper role in this trial, CCTA with CAC will be compared to invasive angiography in over 3,000 patients with stable chest pain and an intermediate pretest probability (10–60%) for CAD.

## Conclusion

An increasing amount of evidence supports the role of CAC testing as a gatekeeper in low-intermediate risk patients with chest pain. A CAC score of zero effectively rules out significant epicardial CAD in low-intermediate risk symptomatic patients (negative predictive value 96–99%) and is associated with a very low risk of future cardiovascular events. Nonetheless, guidelines have yet to endorse the ‘power of zero’ in symptomatic individuals. Overall, the absence of CAC to exclude CAD in low-intermediate risk symptomatic patients appears promising given its exceptional negative predictive value and the test’s ideal gatekeeper characteristics.
